# Neuroprotection of Catalpol for Experimental Acute Focal Ischemic Stroke: Preclinical Evidence and Possible Mechanisms of Antioxidation, Anti-Inflammation, and Antiapoptosis

**DOI:** 10.1155/2017/5058609

**Published:** 2017-07-13

**Authors:** Xia-wei Zheng, Wen-ting Yang, Shuang Chen, Qing-qing Xu, Chun-shuo Shan, Guo-qing Zheng, Ji-chen Ruan

**Affiliations:** ^1^Department of Neurology, The Second Affiliated Hospital and Yuying Children's Hospital of Wenzhou Medical University, Wenzhou 32500, China; ^2^Department of Pediatric Hematology, The Second Affiliated Hospital and Yuying Children's Hospital of Wenzhou Medical University, Wenzhou 325000, China

## Abstract

Neuroprotection is defined as using a therapy that affects the brain tissue in the still-viable ischemic penumbra to salvage or delay the infarction. Catalpol, the main active principle of the root of Radix Rehmanniae, was reported to have pleiotropic neuroprotective effects in neurodegenerative diseases including ischemic stroke. Here, we evaluated the neuroprotective effects of catalpol in experimental acute ischemic stroke. Studies on catalpol in animal models of acute ischemic stroke were identified from 6 databases. Twenty-five studies involving 805 animals were included. Twelve comparisons showed significant effects of catalpol on decreasing infarct size according to 2,3,5-triphenyltetrazolium chloride staining compared with the control (*P* < 0.05). One study reported significant effect of catalpol on reducing infarct size according to magnetic resonance imaging scan compared with the control (*P* < 0.05). Meta-analysis of these studies indicated that catalpol significantly improved the neurological function score according to Zea Longa score, Bederson score, balance beam-walking test, adhesive removal test, bar-grasping score, and corner test compared with the control (*P* < 0.05). In conclusion, catalpol exerted neuroprotective effects for experimental acute focal ischemic stroke, largely through reducing oxidative reactions, inhibiting apoptosis, and repressing inflammatory reactions and autophagy. However, these apparently positive findings should be interpreted with caution because of the methodological flaws.

## 1. Introduction

**Figure 1:**
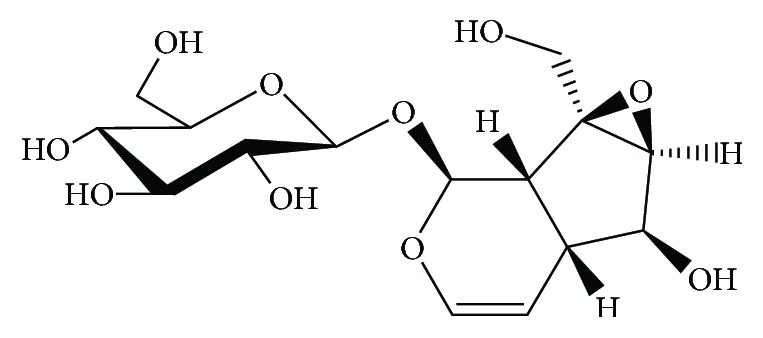
Chemical structure of catalpol.

Neuroprotection refers to the concept of using a therapy that affects the brain tissue in the still-viable ischemic penumbra to salvage or delay the infarction [1, 2]. Possible mechanisms of neuroprotective treatments are to prevent local inflammation, excitotoxicity, free radical damage, neuronal apoptosis, and calcium influx into cells, resulting in both improvement of functional outcomes and reduction of infarct size [3]. In the past decades, a wealth of research has been conducted into the development of numerous neuroprotective treatments capable of reducing brain damage following ischemic stroke of animal models [4]. However, up to now, clinical trials have not identified efficacious neuroprotective therapies for stroke patients [5]. Thus, given the huge translational gap between these animal studies and clinical trials, seeking or developing innovative neuroprotectants is urgently needed. Radix Rehmanniae (Latin name), rehmannia root (English name), Dihuang (Chinese name), the roots of Radix Rehmanniae Recens, was first recorded in the book of *Shennongbencaojing* (*Shennong's Classic of Materia Medica*)—the earliest complete pharmacopoeia of China. In modern times, Radix Rehmanniae and Radix Rehmanniae-based prescriptions are still widely used for treatment of various diseases in China and elsewhere worldwide [6, 7]. Radix Rehmanniae exerts its pharmacological actions on the endocrine system, blood system, immune system, nervous system, cardiovascular system, and so forth [8]. Catalpol (Figure 1), an iridoid glucoside, is the main active principle of the root of Radix Rehmanniae. Recent studies reported that catalpol had pleiotropic neuroprotective effects against hypoxic/ischemic injury, Alzheimer's disease, and Parkinson's disease in both in vivo and in vitro models [9]. Catalpol had been found to have antioxidation, anti-inflammation, antiapoptosis, and other neuroprotective properties [9], suggesting the potential neuroprotective effect of catalpol on stroke [10].

Systematic reviews are considered as the highest level of medical evidence; only data from systematic reviews will be proposed as 1a-evidence according to the levels of evidence from the Centre of Evidence-Based Medicine in Oxford [11]. Preclinical systematic reviews are a novel approach to appraise and synthesize results from animal research into a single and useful document that can indicate the direction for further basic research, reduce and refine the experimental studies, and enhance the rate of success in future clinical trials [12]. However, no systematic analysis has yet been conducted to assess the efficacy of catalpol for experimental ischemic stroke. Therefore, we aimed to identify the current evidence of catalpol as neuroprotective agent in animal models of acute focal ischemic stroke.

## 2. Methods

### 2.1. Search Strategy

Experimental studies of catalpol for acute focal ischemic stroke were identified from PubMed, Web of Science, Excerpta Medica Database (EMBASE), Wanfang Data information site, Chinese National Knowledge Infrastructure (CNKI), and VIP information database. All searches were performed from inception to April 2017. Chinese databases were searched by using the following search terms: “Catalpol” AND [“ischemic stroke” OR “cerebral infarct” OR “middle carotid artery occlusion (MCAO)” OR “cerebral ischemia/reperfusion”]. The term used in English databases was merely “Catalpol.” We manually searched dissertations, conference proceedings, and reference lists of identified publications relevant to this topic.

### 2.2. Eligibility

Experimental studies on catalpol for acute permanent MCAO or temporary MCAO models and compared with vehicle or no treatment were included. Meanwhile, the primary outcome measurements should be neurological function score (NFS) and/or infarct volume (IV). Exclusion criteria were prespecified as follows: (1) the article was a review, case report, comment, only an abstract, or editorial; (2) the article was not an animal study; (3) the article was not a research about acute focal cerebral ischemia model, such as traumatic, global, chronic cerebral ischemic models or not cerebral ischemic models; (4) catalpol was not used as a monothrapy; (5) neither NFS nor IV was used as one of the outcome measurements; (6) there was not a control group in the study; (7) the article was a duplicate publication.

### 2.3. Quality Assessment

The methodological quality of each included study was evaluated by using Collaborative Approach to Meta-Analysis and Review of Animal Data from Experimental Studies (CAMARADES) 10-item checklist [13]: (1) peer-reviewed publication; (2) statements describing control of temperature; (3) randomization to treatment group; (4) allocation concealment; (5) blinded assessment of outcome; (6) avoidance of anesthetics with known notable intrinsic neuroprotective properties; (7) use of animals with relevant comorbidities; (8) sample size calculation; (9) compliance with animal welfare regulations; (10) declared any potential conflict of interest. For calculating an aggregate quality score, each item of this scale was attributed one point. Two authors (ZXW and YWT) independently extracted information and evaluated quality study. Disagreements were solved after discussing the details of the studies.

### 2.4. Data Extraction

The following information of each included study was extracted: (1) the first author's name and publication year, permanent or temporary MCAO, ischemic time, the anesthetic used, and random method; (2) characteristics of animals, including sex, species, weight, and animal number; (3) treatment information, including the drug used, method of treatment, timing for initial treatment, and duration of treatment; (4) outcome measurements, timing for outcome assessments, and corresponding data of mean value, standard deviation, and between-group differences. NFS and/or IV was extracted separately. If outcomes were presented at different time points, we extracted data from the last time point. If studies utilized dose gradient of the drug, we extracted data from the highest dose of catalpol because of the prespecific criteria and the dose-response relationship. If the data were incomplete or presented in graphs, we tried to contact the authors for data needed or calculated using relevant software.

Information of the mechanism studies of catalpol for experimental ischemic stroke among the included articles and other compounds from Rehmanniae Radix was extracted as the following: the first author name, publication year, models used in experiment, interventions in experimental group and control group, observation, and possible mechanisms.

### 2.5. Statistical Analysis

All data of NFS and IV were considered as continuous variables. Meta-analysis was performed with RevMan version 5.0. The random effect model and standard mean difference (SMD) were utilized herein. The *I*^2^ statistics were chosen for the assessment of heterogeneity. Furthermore, to explore potential sources of high heterogeneity, subgroup analyses were performed according to timing for outcome assessments and sex of animals. Difference between groups was determined by partitioning heterogeneity and utilizing the χ^2^ distribution with degrees of freedom (df). When probability value was less than 0.05, the difference was considered significant.

## 3. Results

### 3.1. Study Inclusion

**Figure 2:**
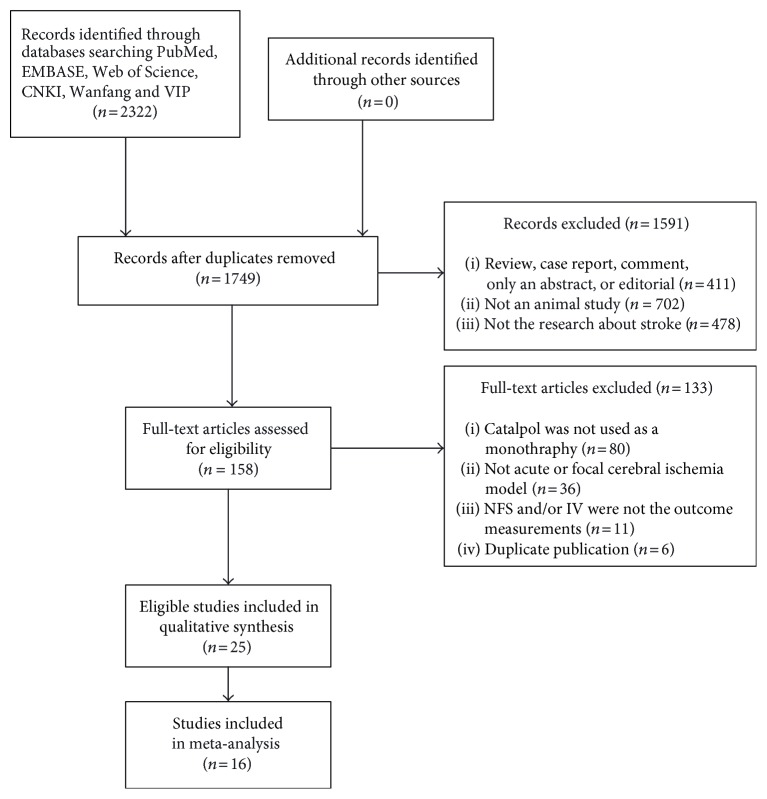
Flow diagram.

We identified 2322 papers after systematical searches of six databases. After removing duplicates, 1749 records remained. By reading the titles and abstracts, 1591 articles were excluded for at least one of the following reasons: (1) the article was a review, case report, comment, only an abstract, or editorial; (2) the article was not an animal study; (3) the article was not a research about cerebral ischemia or stroke. After reviewing the full text of the remaining 158 papers, 80 studies were excluded because catalpol was not used as a monothrapy; 36 studies were excluded because the animal model was not acute focal cerebral ischemia; 11 studies were removed because the outcome measurement was neither NFS nor IV; 6 studies were excluded because they are duplicate publications. Ultimately, 25 eligible articles were identified [14–38] (Figure 2).

### 3.2. Methodological Quality of Included Studies

The quality scores of the 25 included studies ranged from 2 to 7 points. One study [23] got 2 points; 8 studies [16, 19, 20, 24, 28, 30, 34, 35] got 3 points; 11 studies [18, 21, 22, 25–27, 29, 32, 33, 36, 37] got 4 points; one study [15] got 5 points; 3 studies [17, 31, 38] got 6 points; one study [14] got 7 points (Table 1). The average score was 4.00. Eight studies were online Master's thesis or Ph.D. thesis and not formally published. Seven unpublished Master's theses were from Zhou 2008, Xue 2012, Wang 2013, Wang 2015, Min 2015, Qin 2016, and Zhang 2011 [18, 19, 22, 25, 32, 36, 37]. One unpublished Ph.D. thesis was from Liu 2011 [23]. Seven studies described control of temperature [14, 16, 25, 27, 29, 31, 33]. Random allocation to treatment group was described in 21 studies [15, 17–24, 26–34, 36–38], and 2 studies used the method of random digit table [21, 33]. No study reported allocation concealment. Blinded assessment of outcome was described in 4 studies [14, 15, 17, 21, 22]. Twenty-one studies did not use anesthetics with significant intrinsic neuroprotective activity, and the remaining 4 studies did not report the type of anesthetics [15, 16, 23, 24]. No study used animals with relevant comorbidities. One study described the sample size calculation [14]. Eleven studies reported compliance with animal welfare regulations [14–18, 25, 31, 32, 36–38]. Fifteen studies mentioned statement of potential conflict of interests [14, 15, 17–19, 22–25, 31, 32, 35–38].

### 3.3. Study Characteristics

Twenty-five studies with 805 animals were included. Among them, 5 studies [14–17, 38] were published in English and 20 studies were Chinese papers between 2008 and 2016. Twenty-one studies used male and/or female Sprague Dawley (SD) rats; one study [14] used male Wistar rats; four studies [16, 18–20] used Kunming mice. The weight of SD rats used varied from 180 g to 350 g; the weight of Wistar rats used varied from 250 g to 300 g; the weight of mice varied from 22 g to 35 g. Chloral hydrate was used to induce anesthesia in 18 studies, pentobarbital in 2 studies [21, 22], and isoflurane in 1 study [14]; while the remaining 4 studies did not report the type of anesthetics [15, 16, 23, 24]. Nineteen out of the 25 studies utilized permanent MCAO models, and the remaining six studies [14, 21–23, 25, 26] were temporary MCAO models in which ischemic time varied from 1 to 2 hours. Fifteen studies utilized a dose gradient of catalpol: six studies [23, 27–30, 35] administrated 15, 30, and 60 mg kg^−1^ intragastrically, four studies [18, 24, 25, 31] used 1, 5, and 10 mg kg^−1^ intraperitoneally, two studies [21, 22] used 1 and 5 mg kg^−1^ intraperitoneally, one study [32] used 1, 3, and 5 mg kg^−1^ intraperitoneally, one study [36] used 5 and 10 mg kg^−1^ intraperitoneally, and one study [18] used 1.42, 7, and 14.2 mg kg^−1^ intraperitoneally. Twenty-three studies administrated catalpol after stroke; one study administrated catalpol before stroke [14]; and two studies administrated catalpol before and after stroke [18, 25]. In the control group, 14 studies applied same volume of normal saline; 3 studies [23, 28, 36] applied edible oil; one study [14] applied saline; one study [27] applied distilled water; one study [21] applied dimethyl sulfoxide; one study [37] applied 1,2-propylene glycol; 2 studies [29, 35] applied edible oil and normal saline; and 4 studies [20, 22, 26, 38] applied no treatment. Thirteen studies [14, 16, 18–22, 24, 25, 29, 31, 33, 37] adopted IV as outcome measurements; twenty-five studies used NFS as outcome measurements; and 13 studies adopted both above two outcome measurements. However, the methods used to identify IV were different; 11 studies used TTC staining and 2 studies [24, 33] used MRI scan. The standards of NFS were diverse: 13 studies [14, 16, 19–23, 26–29, 35, 37] adopted Zea Longa (ZL) score; 11 studies [15, 17, 18, 23, 27–29, 31, 32, 34, 37] used balance beam-walking test; 7 studies [15, 17, 18, 25, 31, 32, 36] used Bederson score; 4 studies [23, 27, 29, 33] used adhesive removal test; 3 studies [15, 18, 31] used muscular strength test; 3 studies [23, 28, 29] used bar-grasping test; 3 studies [24, 25, 36] used corner test; and 3 studies [26, 34, 38] used cylinder test. From another perspective, neuromuscular function test [18, 25], skilled reaching task test [15, 31], and foot-fault test [33, 38] were utilized in 2 studies. Nerve comprehensive function test [20], stair test [26], tray task box task test [32], grasping test box test [32], real-time gait behavior test [30], and ladder rung walking test were utilized in 1 study. The basic characteristics of the 25 studies were shown in Table 2.

### 3.4. Effectiveness

Eleven studies (15 comparisons) used IV based on TTC staining as outcome measurement. Fourteen comparisons of these studies [14, 16, 18–22, 25, 29, 31, 38] reported that catalpol could significantly reduce IV compared with the control (*P* < 0.05); one comparison [18] showed no significant effect of catalpol for decreasing IV compared with the control, according to TTC staining. Two studies adopted IV based on MRI scan as outcome measurement. One study [33] reported significant effects of catalpol for reducing IV according to MRI scan compared with the control (*P* < 0.05), whereas the other one [24] reported no significance.

**Figure 3:**
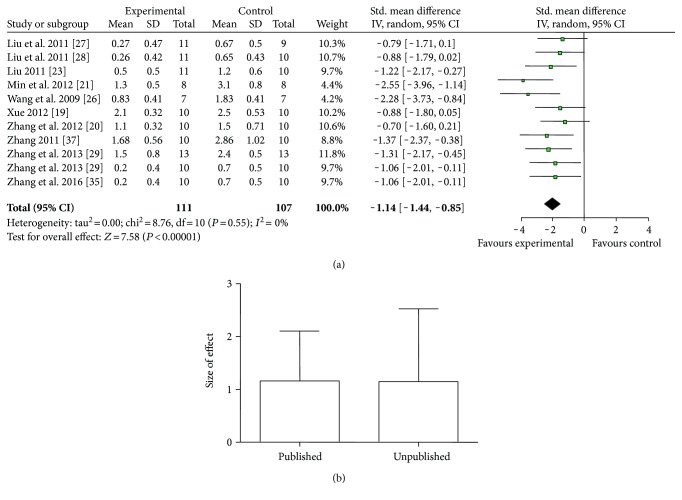
(a) The forest plot: effects of catalpol for improving NFS according to ZL score compared with the control; (b) subgroup analysis of Zea Longa score by published or not.

**Figure 4:**
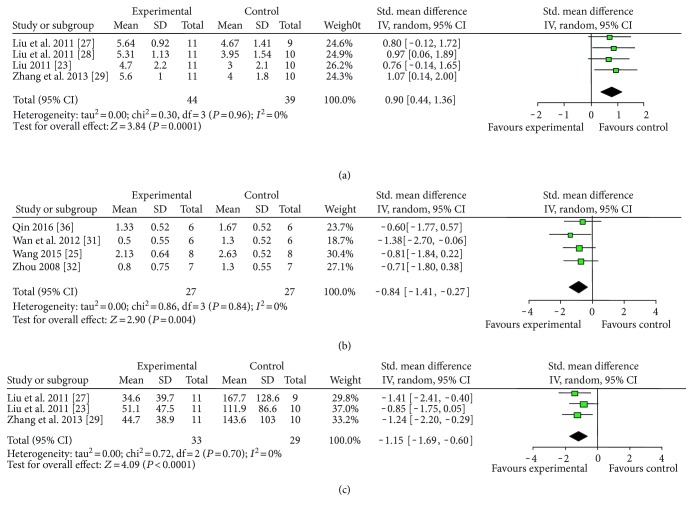
The forest plot: effects of catalpol for improving NFS compared with the control according to (a) balance beam-walking test, (b) Bederson score, and (c) adhesive removal test.

**Figure 5:**
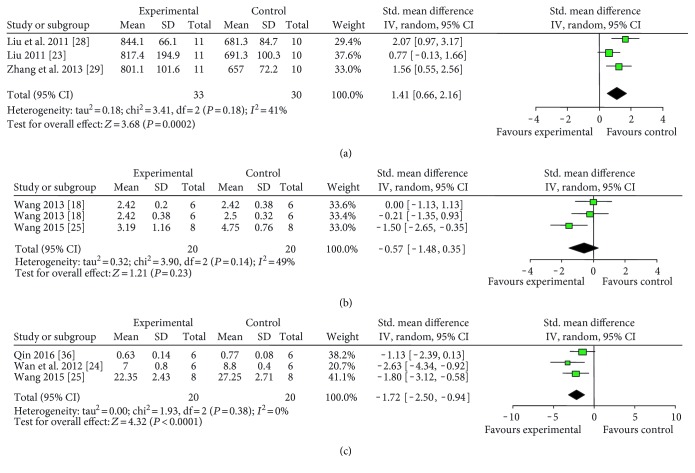
The forest plot: effects of catalpol for improving NFS compared with the control according to (a) bar-grasping test, (b) neuromuscular function test, and (c) corner test.

Various measuring methods of NFS were used as follows: (1) ZL score (13 studies with 15 comparisons): meta-analysis of 11 comparisons [19–21, 23, 26–29, 35, 37] indicated significant effect of catalpol for improving the NFS compared with the control. (*n* = 218, SMD = −1.14, 95% CI: −1.44∼−0.85, *P* < 0.00001; heterogeneity *χ*^2^ = 8.76, df = 10, *P* = 0.55, *I*^2^ = 0%, Figure 3(a)). The remaining four comparisons [14, 16, 22] also showed significance (*P* < 0.05 or *P* < 0.01) but failed to pool analysis due to the absence of primary data. Furthermore, there were three not formally published theses [19, 23, 37] out of 10 studies, which may lead to an inaccurate assessment of the effects of the intervention [39]. Thus, we conducted subgroup analysis and the result showed that there was no difference in effect size between published studies and unpublished studies (Figure 3(b)). (2) Balance beam-walking test (11 studies): meta-analysis of 8 studies [23, 27–29, 31, 32, 34, 37] showed no significant effect of catalpol for improving the NFS compared with the control (*n* = 141, SMD = −1.01, 95% CI: −2.33∼0.31, *P* = 0.14; heterogeneity *χ*^2^ = 68.21, df = 7, *P* < 0.00001; *I*^2^ = 90%). As the value of *I*^2^ was greater than 50%, subgroup analyses were conducted to explore potential sources of high heterogeneity according to timing for outcome assessments. Meta-analysis of two studies [31, 32] in the 21th day subgroup showed that catalpol significantly improved NFS compared with the control (*n* = 26, SMD = −1.22, 95% CI: −1.56∼−0.88, *P* < 0.00001; heterogeneity *χ*^2^ = 1.29, df = 1, *P* = 0.26, *I*^2^ = 23%). Meta-analysis of the 5 studies of the 14th day subgroup indicated that catalpol significantly improved the NFS compared with the control (*n* = 95, SMD = −0.89, 95% CI: −1.33∼−0.45, *P* < 0.0001; heterogeneity *χ*^2^ = 94.06, df = 4, *P* < 0.00001, *I*^2^ = 96%). As the *I*^2^ of latter 14th day subgroup was greater than 50%, we conducted a further subgroup analysis according to animal sex. Meta-analysis of 4 studies [23, 27–29] in male subgroup showed that catalpol significantly improved NFS compared with the control (*n* = 83, SMD = 0.90, 95% CI: 0.44∼1.36, *P* = 0.0001; heterogeneity *χ*^2^ = 0.30, df = 3, *P* = 0.96, *I*^2^ = 0%, Figure 4(a)). Those findings indicated that timing for outcomes assessments and animal sex may be the explanation for the high heterogeneity. Three studies did not provide primary data and thus failed for meta-analysis. Among them, 2 studies [15, 17] reported that catalpol significantly improved NFS (*P* < 0.05), whereas one study [18] showed no significance. (3) Bederson score (7 studies): meta-analysis of 4 studies [25, 31, 32, 36] showed significant effect of catalpol for improving NFS according to Bederson score compared with control (*n* = 54, SMD = −0.84, 95% CI: −1.41∼−0.27, *P* = 0.004; heterogeneity *χ*^2^ = 0.84, df = 3, *P* = 0.84; *I*^2^ = 0%, Figure 4(b)). Three studies failed to pool analysis due to the absence of primary data. Among which, 2 studies [15, 18] reported that the effect of catalpol on NFS was significant (*P* < 0.05), but one study [17] showed no significance. (4) Adhesive removal test (4 studies): meta-analysis of 3 studies [23, 27, 29] indicated that catalpol significantly improved NFS compared with the control according to adhesive removal test (*n* = 62, SMD = −1.15, 95% CI: −1.69∼−0.60, *P* < 0.0001; heterogeneity *χ*^2^ = 0.72, df = 2, *P* = 0.70; *I*^2^ = 0%, Figure 4(c)). One study [33] also reported the significance (*P* < 0.05) but failed for pool analysis due to the absence of primary data. (5) Bar-grasping test: meta-analysis of 3 studies [23, 28, 29] indicated significant effects of catalpol for improving NFS compared with the control (*n* = 63, SMD = 1.41, 95% CI: 0.66∼2.16, *P* = 0.0002; heterogeneity *χ*^2^ = 3.41, df = 2, *P* = 0.18; *I*^2^ = 41%, Figure 5(a)). (6) Neuromuscular function test (2 studies with 3 comparisons): meta-analysis of 3 comparisons [18, 25] showed no significant effect of catalpol for improving NFS according to neuromuscular function test (*n* = 40, SMD = −0.57, 95% CI: −1.48∼0.35, *P* = 0.23; heterogeneity *χ*^2^ = 3.90, df = 2, *P* = 0.14; *I*^2^ = 49%, Figure 5(b)). (8) Corner test (3 studies): meta-analysis of 3 studies [24, 25, 36] showed significant effect of catalpol for improving NFS (*n* = 40, SMD = −1.72, 95% CI: −2.50∼−0.94, *P* < 0.0001; heterogeneity *χ*^2^ = 1.93, df = 2, *P* = 0.38; *I*^2^ = 0%, Figure 5(c)). (9) Others: several studies showed significant effects of catalpol for reducing NFS compared with control (*P* < 0.05 or *P* < 0.01) according to cylinder test [26, 34, 38], skilled reaching task test [31], neural comprehensive function test [20], stair test [26], tray task box task test [32], grasping test box test [32], foot-fault test [33, 38], Ladder rung walking test [36], and real-time gait behavior test [30], respectively; one study [15] showed no significance according to skilled reaching task test; three studies showed no significance according to muscular strength test [15, 18, 31].

### 3.5. Neuroprotective Mechanisms of Catalpol for Ischemic Stroke

The mechanisms of neuroprotection of catalpol on ischemic stroke were studied in a total of 19 included articles [14, 15, 17, 18, 20–25, 27, 28, 31–36, 38] and summarized as follows: (1) reduction of oxidative reactions by increasing the activity of SOD, GSH-PX, and catalase, increasing the expression of NOX2 and decreasing the concentration of MDA and NO [20–23]; (2) inhibition of apoptosis by increasing bcl-2 expression and decreasing the expression of cleaved caspase-3, caspase-9, and Bax [22, 23, 36]; (3) repression of inflammatory reactions by decreasing the expression of IL10 [23]; (4) repression of autophagy by increasing LC3 expression [25]; (5) relief of energy exhaustion by decreasing the content of lactic acid, increasing the content of pyruvic acid, and improving the activity of Na^+^, K^+^-ATPase and Ca^2+^, Mg^2+^-ATPase [23, 27]; (6) promotion of survival, reparation, and regeneration of neural cells through increasing the expression of NGF, BDNF, VEGF, bFGF, TrKA, TrkB, AKt, and PI3K, increasing the mRNA levels of NGF, BDNF, TrKA, and AKt, and decreasing CDNF expression and PI3K mRNA [15, 23, 24, 28, 31, 33–35]; (7) enhancement of angiogenesis by increasing the expression of EPO, EPOR, VEGF, JAK2, pJAK2, STAT3, and Ang-1 [17, 18, 31–34, 38]; (8) neuroprotection through GLP-1R/*β*-endorphin pathway [14]. Characteristics of mechanism studies of catalpol on experimental ischemic stroke were showed in Table 3.

Currently, more than 140 compounds are being isolated from Radix Rehmanniae, including rehmaglutoside E, ajugol, leucosceptoside A, jionoside D, acteoside, salidrosid, jionoside B1, vanillin, oleanolic acid, and geniposide [40–42]. Liu [42] studied the chemical compounds of *Rehmannia glutinosa Libosch* and assayed their bioactivities, indicating that rehmaglutoside E, 6-O-E-caffeoyl ajugol, leucosceptoside A, and jionoside D had antioxidation activities and acteoside, salidrosid, leucosceptoside A, jionoside D, jionoside B1, and vanillin had anti-inflammation activities. Ajugol was reported to repress inflammatory reactions by decreasing the NO in lipopolysaccharide- (LPS-) induced mouse microglial cells [43]. Oleanolic acid attenuated inflammation in bile duct-ligated SD rats through decreasing serum TNF-*α*, IL-1*β*, and IL-6 [44] and attenuated oxidative reactions in cardiac toxicity SD rats by decreasing activities of GSH, SOD, and catalase and attenuating the level of MDA [45]. Liu et al. [46] demonstrated that geniposide exerted antioxidation activities by increasing the expression of Bcl-2 and HO-1 in PC12 cells induced by hydrogen peroxide. Besides, geniposide was reported to decrease the secretion of TNF-*α*, IL-1*β*, IL-6, IL-8, and IL-10 in primary microglial cell oxygen-glucose deprivation/4h model, showing anti-inflammation activities [47]. Characteristics of those mechanism studies were showed in Table 4.

## 4. Discussion

### 4.1. Efficacy of Catalpol

To our knowledge, it is the first systematic review that investigated the efficacy of catalpol for experimental acute focal ischemic stroke. Our analysis of 25 studies with 805 animals showed that catalpol significantly reduced IV and improved NFS, suggesting the potential neuroprotective functions of catalpol in experimental acute focal ischemic stroke. However, given the methodological flaws, the overall available evidence from the present study should be interpreted cautiously.

### 4.2. Limitations

Some limitations should be considered while interpreting our study. First, we only included studies from Chinese and English databases. The absence of studies written in other languages may, to a certain degree, generate selective bias [48]. Second, only 5 out of 25 studies were English papers and the remaining ones were all Chinese papers, thereby limiting generalization of the findings. Third, the quality scores ranging from 2 to 7 points revealed low methodological quality of included studies. Most of the research had flaws in aspects of randomization, allocation concealment, and blinding and sample size calculation, which are the core standards of study design [49]. In addition, none of the included studies used animals with relevant comorbidities, which would have created more relevant models for human pathology [49]. Thus, the present study should be interpreted cautiously.

### 4.3. Implications

There is a wealth of evidence showing the poor design of animal research [50], which is considered as a roadblock to translate animal research into promising preclinical drug treatments for human disease [51]. In the present study, the low quality of included studies rests with inherent limitations in the primary studies. Thus, some measurements have been developed to directly or indirectly overcome methodology quality issues for animal researches. The animal research: reporting in vivo experiments (ARRIVE) [52] is a reporting guideline consisting of a 20-item checklist for the Introduction, Methods, Results, and Discussion. We recommend to use the ARRIVE guidelines when designing animal research on catalpol, in order to improve the methodological quality. The Stroke Therapy Academic Industry Roundtable (STAIR) meetings [53] provide recommendations on dose, time window, design, outcome assessment, animal species, and model of preclinical studies of acute stroke. We also suggest utilizing the STAIR recommendations specifically for the study of catalpol treatment for experimental stroke.

It is disappointing that many drugs that showed significant effects and looked promising in animal researches failed to translate into clinical drug treatments [54]. The application of excessive drug doses and the timing of drug administration in animal models, which are inapplicable for human disease, are considered to be two of the main reasons for the failure to translate from animal models to human [54]. In the present systematic review, doses of catalpol and timing for initial administration in animal models were inconsistent among the 25 included studies. Thus, we suggest further studies to determinate the optimal gradient doses and timing of administration in animal models of acute ischemic stroke.

The molecular and biological mechanisms of the neuroprotective effects of catalpol have not been fully elucidated. The present study showed that catalpol had neuroprotective effects for ischemic stroke through different mechanisms as follows: (1) reduction of oxidative reactions by increasing the activity of SOD, GSH-PX, and catalase, increasing the expression of NOX2 and decreasing the concentration of MDA and NO; (2) inhibition of apoptosis by increasing bcl-2 expression and decreasing the expression of cleaved caspase-3, caspase-9, and Bax; (3) repression of inflammatory reactions by decreasing the expression of IL10; (4) repression of autophagy by increasing LC3 expression; (5) relief of energy exhaustion by decreasing lactic acid content, increasing pyruvic acid content, and improving the activity of Na^+^, K^+^-ATPase and Ca^2+^, Mg^2+^-ATPase; (6) promotion of survival, reparation, and regeneration of neural cells through upregulating the expression of VEGF, bFGF, TrKA, TrkB, AKt, and PI3K; (7) enhancement of angiogenesis by upregulating the expression of EPO, EPOR, VEGF, JAK2, pJAK2, STAT3, and Ang-1; (8) neuroprotection through GLP-1R/*β*-endorphin pathway. Besides, other compounds from Radix Rehmanniae were reported to have antioxidation, anti-inflammation, and antiapoptosis activities. However, the efficacy of catalpol in terms of cellular and molecular alteration mechanisms along with functional improvement is worthy of further studies.

A total of 18 measuring methods for NFS were used in the 25 included studies, which indicated that the measuring methods for NFS were diverse and inconsistent. Whether and how the different measuring methods for NFS would affect the result of animal studies of acute ischemic stroke is expected to be further studied. Moreover, it is necessary to explore the accuracy of different measuring methods for NFS to filtrate optimum standards for NFS.

## 5. Conclusion

The present study demonstrated that catalpol could improve NFS and reduce IV, exerting potential neuroprotective effects on experimental acute focal ischemic stroke, mainly through reducing oxidative reaction, inhibiting apoptosis, and repressing inflammatory reactions and autophagy. In addition, catalpol may be a promising candidate for clinical trials. Future rigor-randomized controlled trials are needed.

## Figures and Tables

**Table 1:** Quality assessment of included studies.

Study (years)	1	2	3	4	5	6	7	8	9	10	Total
Jia et al. [14]	+	+	−	−	+	+	−	+	+	+	7
Wan et al. [15]	+	−	+	−	+	NR	−	−	+	+	5
Wan et al. 2013 [16]	+	+	−	−	−	NR	−	−	+	−	3
Zhu et al. [17]	+	−	+	−	+	+	−	−	+	+	6
Wang [18]	−	−	+	−	−	+	−	−	+	+	4
Xue [19]	−	−	+	−	−	+	−	−	−	+	3
Zhang et al. [20]	+	−	+	−	−	+	−	−	−	−	3
Min et al. [21]	+	−	+	−	+	+	−	−	−	−	4
Min [22]	−	−	+	−	+	+	−	−	−	+	4
Liu 2011 [23]	−	−	+	−	−	NR	−	−	−	+	2
Wan et al. 2012 [24]	+	−	+	−	−	NR	−	−	−	+	3
Wang [25]	−	+	−	−	−	+	−	−	+	+	4
Wang et al. [26]	+	−	+	−	+	+	−	−	−	−	4
Liu et al. 2011 [27]	+	+	+	−	−	+	−	−	−	−	4
Liu et al. 2011 [28]	+	−	+	−	−	+	−	−	−	−	3
Zhang et al. 2013 [29]	+	+	+	−	−	+	−	−	−	−	4
Zhang et al. 2013 [30]	+	−	+	−	−	+	−	−	−	−	3
Wan et al. 2012 [31]	+	+	+	−	−	+	−	−	+	+	6
Zhou [32]	−	−	+	−	−	+	−	−	+	+	4
Wan et al. 2013 [33]	+	+	+	−	−	+	−	−	−	−	4
Tan et al. [34]	+	−	+	−	−	+	−	−	−	−	3
Zhang et al. [35]	+	−	−	−	−	+	−	−	−	+	3
Qin [36]	−	−	+	−	−	+	−	−	+	+	4
Zhang [37]	−	−	+	−	−	+	−	−	+	+	4
Wan et al. [38]	+	−	+	−	+	+	−	−	+	+	6

1: peer-reviewed publication; 2: statements describing control of temperature; 3: randomization to treatment group; 4: allocation concealment; 5: blinded assessment of outcome; 6: avoidance of anesthetics with known notable intrinsic neuroprotective properties; 7: use of animals with relevant comorbidities; 8: sample size calculation; 9: compliance with animal welfare regulations; 10: declared any potential conflict of interest; NR: not reported.

**Table 2:** Basic characteristics of the included studies.

Study (years)	Species (sex, *n* = experimental/control group)	Weight	Random method	Model (method)	Anesthetic	Method of administration		Outcome index (time)	Intergroup differences
						Experimental group	Control group		
Jia et al. [14]	Wistar rats (male, 6/6)	250–300 g	Not mention random	MCAO/1 h	1.0% isoflurane	Catalpol (98%, 100 *μ*g, icv); 15 min before occlusion	Saline (same volume, icv); 15 min before occlusion	(1) NFS (ZL score, 2 d) (2) IV (TTC, 2 d)	(1) *P* < 0.05 (2) *P* < 0.05
	Wistar rats (male, 7/7)	250–300 g	Not mention random	MCAO/1 h	1.0% isoflurane	Catalpol (98%, 100 *μ*g, icv.) and saline (5 *μ*L,icv); 15 min before occlusion	Saline (same volume, icv); 15 min before occlusion	(1) IV (TTC, 2 d)	(1) *P* < 0.05
Wan et al. [15]	SD rats (male, 10/10)	220–280 g	Not mention method	Permanent MCAO	NR	Catalpol (5 mg kg^−1^, ip); 24 h after occlusion; once daily for 7 d	Normal saline (same volume, ip); 24 h after occlusion; once daily for 7 d	(1) NFS (Bederson score, 1, 4, 7, and 15 d) (2) NFS (muscular strength test, 1, 4, 7, and 15 d) (3) NFS (balance beam-walking test, 1, 4, 7, and 15 d) (4) NFS (skilled reaching task test, 1, 4, 7, and 15 d) (5) GAP43 express (6) GAP43-positive cell count (7) Synapse count	(1) *P* > 0.05 (2) *P* > 0.05 (3) *P* < 0.05 (4) *P* > 0.05 (5) *P* < 0.05 (6) *P* < 0.05 (7) *P* < 0.05
Wan et al. 2013 [16]	Kunming mice (both, 10/10)	25–30 g	Not mention random	Permanent MCAO	NR	Catalpol (9 mg kg^−1^, ip); 24 h after occlusion; once daily for 3 d	Normal saline (same volume, ip); 24 h after occlusion; once daily for 3 d	(1) NFS (ZL score, 1, 2, and 3 d) (2) IV (TTC, 3 d) (3) Cerebral blood flow ratio	(1) *P* < 0.01 (2) *P* < 0.05 (3) *P* < 0.05
Zhu et al. [17]	SD rats (male, 24/24)	220–280 g	Not mention method	Permanent MCAO	Chloral hydrate	Catalpol (5 mg kg^−1^, ip); 24 h after occlusion; once daily for 7 d	Normal saline (same volume, ip); 24 h after occlusion; once daily for 7 d	(1) NFS (Bederson score, 1, 4, 7, and 15 d) (2) NFS (balance beam-walking test, 1, 4, 7, and 15 d) (3) VWF and PCNA colocalization point count (4) EPO expression (5) VEGF expression (6) EPO-positive cell (7) VEGF-positive cell (8) Vascular pattern (9) Brain capillary endothelial cell microstructure	(1) *P* < 0.05 (2) *P* < 0.05 (3) *P* < 0.05 (4) *P* < 0.05 (5) *P* < 0.05 (6) *P* < 0.05 (7) *P* < 0.05 (8) NR (9) NR
Wang [18]	Kunming mice (NR, 6/6)	23–28 g	Not mention method	Permanent MCAO	3.5% chloral hydrate (10 mL kg^−1^)	Catalpol (1.42, 7, and 14.2 mg kg^−1^, ip); after occlusion; once daily for 3 d	Normal saline (same volume, ip); after occlusion; once daily for 3 d	(1) NFS (neuromuscular function test, 3 d) (2) NFS (muscular strength test, 3 d) (3) IV (TTC, 3 d)	(1) *P* > 0.05 (2) *P* > 0.05 (3) *P* < 0.05
	Kunming mice (NR, 6/6)	23–28 g	Not mention method	Permanent MCAO	3.5% chloral hydrate (10 mL kg^−1^)	Catalpol (14.2 mg kg^−1^, ip); 0.5 h before, 1 h after or 24 h after occlusion; once daily for 3 d	Normal saline (same volume, ip); after occlusion; once daily for 3 d	(1) NFS (neuromuscular function test, 3 d) (2) NFS (muscular strength test, 3 d) (3) IV (TTC, 3 d)	(1) *P* > 0.05 (2) *P* > 0.05 (3) *P* > 0.05
	SD rats (NR, 8/8)	250–300 g	Not mention method	Permanent MCAO	3.5% chloral hydrate (10 mL kg^−1^)	Catalpol (1, 5, and 10 mg kg^−1^, ip); after occlusion; once daily for 7 d	Normal saline (same volume, ip); after occlusion; once daily for 7 d	(1) NFS (Bederson score, 1, 4, 7, 15, 21, and 28 d) (2) NFS (muscular strength test, 1, 4, 7, 15, 21, and 28 d) (3) NFS (balance beam-walking test, 1, 4, 7, 15, 21, 28 d) (4) IV (TTC, 3 d)	(1) *P* > 0.05 (2) *P* > 0.05 (3) *P* > 0.05 (4) *P* > 0.05
Xue [19]	Kunming mice (both, 10/10)	22–28 g	Not mention method	Permanent MCAO	Chloral hydrate	Catalpol (9 mg kg^−1^, iv); 24 h after occlusion; once daily for 3 d	Normal saline (same volume, iv); 24 h after occlusion; once daily for 3 d	(1) NFS (ZL score, 1, 2, and 3 d) (2) IV (TTC, 3 d) (3) Cerebral blood flow ratio (4) Hippocampal tissue morphology	(1) *P* < 0.01 (2) *P* < 0.01 (3) *P* < 0.01 (4) NR
Zhang et al. [20]	Kunming mice (male, 10/10)	25–35 g	Not mention method	Permanent MCAO	Chloral hydrate (350 mg kg^−1^)	Catalpol (9 mg kg^−1^, iv); 3 h after occlusion; once daily for 7 d	MCAO without any intervention	(1) NFS (ZL score, 1, 4, and 7 d) (2) NFS (nerve comprehensive function test, 1, 4, and 7 d) (3) IV (TTC, 8 d)	(1) *P* < 0.05 (2) *P* < 0.05 (3) *P* < 0.05
Min et al. [21]	SD rats (NR, 8/8)	260–280 g	Random digit table	MCAO/2 h	Pentobarbital (60 mg kg^−1^)	Catalpol (1 and 5 mg kg^−1^, ip); 2 h after occlusion	Dimethyl sulfoxide (0.1 mol kg^−1^, ip); 2 h after occlusion	(1) NFS (ZL score, 2 d) (2) IV (TTC, 2 d)	(1) *P* < 0.05 (2) *P* < 0.05
Min [22]	SD rats (male, 6/6)	280–320 g	Not mention method	MCAO/2 h	Pentobarbital (60 mg kg^−1^)	Catalpol (1 and 5 mg kg^−1^, ip); 2 h after occlusion	MCAO without any intervention	(1) NFS (ZL score, 2 d) (2) IV (TTC, 2 d)	(1) *P* < 0.05 (2) *P* < 0.05
	SD rats (male, 6/6)	280–320 g	Not mention method	MCAO/2 h	Pentobarbital (60 mg kg^−1^)	Catalpol (1 and 5 mg kg^−1^, ip); 2 h after occlusion	MCAO without any intervention	(1) NFS (ZL score, 2 d) (2) IV (TTC, 2 d)	(1) *P* < 0.05 (2) *P* < 0.05
Liu 2011 [23]	SD rats (male, 11/10)	260–280 g	Not mention method	MCAO/2 h	NR	Catalpol (15, 30, and 60 mg kg^−1^, ig); 2 d after occlusion; once daily for 12 d	Edible oil (same volume, ig); 2 d after occlusion; once daily for 12 d	(1) NFS (ZL score, 3, 7, 10, and 14 d) (2) NFS (balance beam-walking test, 3, 7, 10, and 14 d) (3) NFS (adhesive removal test, 3, 7, 10, and 14 d) (4) NFS (bar-grasping test, 3, 7, 10, and 14 d) (5) Lactic acid content (6) Pyruvic acid content (7) Lactic acid content/pyruvic acid content (8) Na^+^, K^+^-ATPase activity (9) Ca^2+^, Mg^2+^-ATPase activity	(1) *P* < 0.01 (2) *P* < 0.05 (3) *P* < 0.05 (4) *P* < 0.05 (5) *P* < 0.05 (6) *P* < 0.01 (7) *P* < 0.01 (8) *P* < 0.01 (9) *P* < 0.01
Wan et al. 2012 [24]	SD rats (both, 6/6)	220–250 g	Not mention method	Permanent MCAO	NR	Catalpol (1, 5, and 10 mg kg^−1^, ip); 24 h after occlusion; once daily for 7 d	Normal saline (same volume, ip); 24 h after occlusion; once daily for 7 d	(1) NFS (corner test, 1, 4, 7, and 15 d) (2) IV (MRI, 1 and 15 d) (3) Dendritic branch (4) Spine density (5) P38 IOD	(1) *P* < 0.05 (2) *P* > 0.05 (3) *P* < 0.05 (4) *P* < 0.05 (5) *P* < 0.05
Wang [25]	SD rats (NR, 8/8)	250–350 g	Not mention random	MCAO/2 h	3.5% chloral hydrate (10 mL kg^−1^)	Catalpol (1, 5, and 10 mg kg^−1^, ip); 12 h before and 1 h after occlusion	Normal saline (same volume, ip); 12 h before and 1 h after occlusion	(1) NFS (Bederson score, 1 d) (2) NFS (neuromuscular function test, 1 d) (3) NFS (corner test, 1 d) (4) IV (TTC, 1 d) (5) LC3 express	(1) *P* > 0.05 (2) *P* < 0.01 (3) *P* < 0.05 (4) *P* < 0.05 (5) NR
Wang et al. [26]	SD rats (male, 6/6)	220–280 g	Not mention method	MCAO/1.5 h	3.5% chloral hydrate (10 mL kg^−1^)	Catalpol (5 mg kg^−1^, ip); 1.5 h after occlusion; once daily for 7 d	MCAO without any intervention	(1) NFS (ZL score, 1, 4, and 7 d) (2) NFS (stair test, 1, 4, and 7 d) (3) NFS (cylinder test, 1, 4, and 7 d)	(1) *P* < 0.05 (2) *P* < 0.05 (3) *P* < 0.05
Liu et al. 2011 [27]	SD rats (male, 12/14)	260–290 g	Not mention method	Permanent MCAO	10% chloral hydrate (350 mg kg^−1^)	Catalpol (15, 30, and 60 mg kg^−1^, ig); 2 d after occlusion; once daily for 12 d	Distilled water (same volume, ig); 2 d after occlusion; once daily for 12 d	(1) NFS (ZL score, 3,6, 9, 12, and 14 d) (2) NFS (balance beam-walking test, 3, 9, and 14 d) (3) NFS (adhesive removal test, 3, 9, and 14 d) (4) Lactic acid content (5) Pyruvic acid content (6) Lactic acid/pyruvic acid (7) Na^+^, K^+^-ATPase activity (8) Ca^2+^, Mg^2+^-ATPase activity	(1) *P* < 0.05 (2) *P* < 0.05 (3) *P* < 0.01 (4) *P* < 0.05 (5) *P* < 0.05 (6) *P* < 0.01 (7) *P* < 0.01 (8) *P* < 0.01
Liu et al. 2011 [28]	SD rats (male, 11/10)	260–290 g	Not mention method	Permanent MCAO	10% chloral hydrate (350 mg kg^−1^)	Catalpol (15, 30, and 60 mg kg^−1^, ig); 2 d after occlusion; once daily for 12 d	Edible oil (same volume, ig); 2 d after occlusion; once daily for 12 d	(1) NFS (ZL score, 7, 10, and 14 d) (2) NFS (balance beam-walking test, 7, 10, and 14 d) (3) NFS (bar-grasping test, 7, 10, and 14 d) (4) NGF IOD (5) BDN IOD (6) NGF mRNA express (7) BDNF mRNA express	(1) *P* < 0.05 (2) *P* < 0.05 (3) *P* < 0.01 (4) *P* > 0.05 (5) *P* < 0.01 (6) *P* > 0.05 (7) *P* < 0.01
Zhang et al. 2013 [29]	SD rats (male, 13/13)	210–240 g	Not mention method	Permanent MCAO	10% chloral hydrate (350 mg kg^−1^)	Catalpol (15, 30, and 60 mg kg^−1^, ig); after occlusion	Normal saline (same volume, ig); after occlusion	(1) NFS (ZL score, 6 and 24 h) (2) IV (TTC, 24 h) (3) Brain water content (4) Water content	(1) *P* < 0.001 (2) *P* > 0.05 (3) *P* > 0.05 (4) *P* < 0.05
	SD rats (male, 10/10)	260–290 g	Not mention method	Permanent MCAO	10% chloral hydrate (350 mg kg^−1^)	Catalpol (15, 30, and 60 mg kg^−1^, ig); 2 d after occlusion; once daily for 12 d	Edible oil (same volume, ig) and normal saline (same volume, ip); 2 d after occlusion; once daily for 12 d	(1) NFS (ZL score, 3, 7, 10, and 14 d) (2) NFS (balance beam-walking test, 3, 7, 10, and 14 d) (3) NFS (adhesive removal test, 3,7,10, and 14 d) (4) NFS (bar-grasping test, 3, 7, 10, and 14 d) (5) Normal neuron count (6) Nissl body iod (7) IL-6 content (8) IL-10 content (9) NF-kBp65 content (10) Cerebral cortex ultrastructure	(1) *P* < 0.05 (2) *P* < 0.05 (3) *P* < 0.01 (4) *P* < 0.01 (5) *P* < 0.05 (6) *P* < 0.05 (7) *P* < 0.01 (8) *P* > 0.05 (9) *P* > 0.05 (10) NR
Zhang et al. 2013 [30]	SD rats (male, 18/18)	260–290 g	Not mention method	Permanent MCAO	10% chloral hydrate (3.5 mL kg^−1^)	Catalpol (15, 30, and 60 mg kg^−1^, ig); 6 h after occlusion; once daily for 14 d	Normal saline (same volume, ig); 6 h after occlusion; once daily for 14 d	(1) NFS (real-time gait behavior test, duty cycle, 15 d) (2) NFS (real-time gait behavior test, four feet swinging time, 15 d) (3) NFS (real-time gait behavior test, four feet supporting time, 15 d) (4) NFS (real-time gait behavior test, walking speed, 15 d) (5) NFS (real-time gait behavior test, the average of body angle, 15 d) (6) NFS (real-time gait behavior test, the absolute value of body angle, 15 d) (7) NFS (real-time gait behavior test, coordination index, 15 d) (8) NFS (real-time gait behavior test, several feet supporting time, 15 d)	(1) *P* < 0.05(A, B, LFL RF) *P* > 0.05(LB) (2) *P* > 0.05(LF, RB) *P* < 0.05(RF) *P* < 0.01(LB) (3) *P* > 0.05(LF, LB) *P* < 0.05(RF) *P* < 0.01(RB) (4) *P* > 0.05 (5) *P* > 0.05 (6) *P* < 0.01 (7) *P* < 0.05(RF-LB, F-RB, RF-LF) (8) *P* > 0.05(LF&RB, LB&RF, LF&LB&RF)
Wan et al. 2012 [31]	SD rats (male, 9/9)	220–250 g	Not mention method	Permanent MCAO	3.5% chloral hydrate (10 mL kg^−1^)	Catalpol (1, 5, and 10 mg kg^−1^, ip); 6 h after occlusion; once daily for 7 d; catalpol (5 mg kg^−1^, ip); 24 h after occlusion; once daily for 7 d	Normal saline (same volume, ip); 6 h after occlusion; once daily for 7 d	(1) NFS (Bederson score, 1, 4, 7, 15, and 21 d) (2) NFS (muscle strength test, 1, 4, 7, 15, and 21 d) (3) NFS (balance beam-walking test, 1, 4, 7, 15, and 21 d) (4) NFS (skilled reaching task test, 1, 4, 7, 15, and 21 d) (5) IV (TTC, 3 d) (6) GAP-43-positive cell count (7) GAP-43 expression	(1) *P* < 0.05 (2) *P* > 0.05 (3) *P* < 0.05 (4) *P* < 0.05 (5) *P* < 0.01 (6) *P* < 0.05 (7) *P* < 0.05
Zhou [32]	SD rats (both, 7/7)	250–300 g	Not mention method	Permanent MCAO	3.5% chloral hydrate (10 mL kg^−1^)	Catalpol (1, 3, and 5 mg kg^−1^, ip); 24 h after occlusion; once daily for 7 d	Normal saline (same volume, ip); 24 h after occlusion; once daily for 7 d	(1) NFS (Bederson score, 2, 4, 7, 15, and 21 d) (2) NFS (tray task box task test, 2, 4, 7, 15, and 21 d) (3) NFS (grasping test box test, 2, 4, 7, 15, and 21 d) (4) NFS (balance beam-walking test, 2, 4, 7, 15, and 21 d)	(1) *P* < 0.05 (2) *P* < 0.05 (3) *P* < 0.05 (4) *P* < 0.05
Wan et al. 2013 [33]	SD rats (both, 6/6)	220–250 g	Random digit table	Permanent MCAO	Chloral hydrate	Catalpol (5 mg kg^−1^, ip); 24 h after occlusion; once daily for 7 d	Normal saline (same volume, ip); 24 h after occlusion; once daily for 7 d	(1) NFS (adhesive removal test, 1, 4, 7, 14, 21, and 28 d) (2) NFS (foot-fault test, 1, 4, 7, 14, 21, and 28 d) (3) IV (MRI, 1 and 28 d) (4) Midline-crossing CST fiber (5) CST axonal sprouting	(1) *P* < 0.05 (2) *P* < 0.05 (3) *P* > 0.05 (4) *P* < 0.05 (5) *P* < 0.05
Tan et al. [34]	SD rats (NR, 6/6)	200–220 g	Not mention method	Permanent MCAO	3.5% chloral hydrate	Catalpol (5 mg kg^−1^, ip); 6 h after occlusion; once daily for 7 d	Normal saline (same volume, ip); 6 h after occlusion; once daily for 7 d	(1) NFS (balance beam-walking test, 1, 4, 7, and 14 d) (2) NFS (cylinder test, 1, 4, 7, and 14 d) (3) Vessel length (4) Neuron count (5) Glial cell count (6) Cell morphology	(1) *P* < 0.01 (2) *P* < 0.01 (3) *P* < 0.01 (4) *P* < 0.05 (5) *P* < 0.05 (6) NR
Zhang et al. [35]	SD rats (male, 10/10)	200–250 g	Not mention random	Permanent MCAO	Chloral hydrate (300 mg kg^−1^)	Catalpol (15, 30, 60 mg kg^−1^, ig); 3 d after occlusion; once daily for 12 d	Edible oil (same volume, ig) and normal saline (same volume, ip); 3 d after occlusion; once daily for 12 d	(1) NFS (Zea Longa score, 3, 7, 10, and 14 d) (2) LFB IOD (3) MBP IOD (4) Brain pathohistology	(1) *P* < 0.05 (2) *P* < 0.01 (3) *P* < 0.01 (4) NR
Qin [36]	SD rats (male, 6/6)	220–280 g	Not mention method	Permanent MCAO	3.5% chloral hydrate	Catalpol (5 and 10 mg kg^−1^, ip); 6 h after occlusion; once daily for 21 d	Normal saline (100 g/1 mL, ip); 6 h after occlusion; once daily for 21 d	(1) NFS (Bederson score, 1, 3, 7, 14, and 21 d) (2) NFS (corner test, 1, 3, 7, 14, and 21 d) (3) NFS (ladder rung walking test, 1, 3, 7, 14, and 21 d)	(1) *P* > 0.05 (2) *P* > 0.05 (3) *P* < 0.01 (4) *P* > 0.05
Zhang [37]	SD rats (male, 10/10)	180–220 g	Not mention method	Permanent MCAO	Chloral hydrate	Catalpol (NR, iv); 3 h after occlusion; once daily for 7 d	1, 2-Propylene glycol (NR, iv); 3 h after occlusion; once daily for7 d	(1) NFS (Zea Longa score, 7 d) (2) NFS (balance beam-walking test score, 7 d) (3) IV (TTC, 7 d)	(1) *P* < 0.01 (2) *P* < 0.01 (3) *P* < 0.05
Wan et al. [38]	SD rats (male, 9/9)	220–250 g	Not mention method	Permanent MCAO	Chloral hydrate	Catalpol (5 mg kg^−1^, ip); 1 d after occlusion; once daily for 7 d	MCAO without any intervention	(1) NFS (cylinder test, 1, 4, 7, and 15 d) (2) NFS (foot-fault test, 1, 4, 7, and 15 d) (3) Cerebral blood flow ratio (4) VWF-PCNA colocalization number (5) VWF-PCNA colocalization area IOD (6) pSTAT3 translocation number (7) pSTAT3-positive cell IOD (8) EPO/NADPH IOD (9) EPOR/GAPDH IOD (10) pJAK2/NADPH IOD (11) pSTAT3/GAPDH IOD (12) pSTAT3-VEGF DNA-binding activity (13) VEGF mRNA (14) VEGF/NADPH IOD (15) VEGF-positive cell IOD	(1) *P* < 0.01 (2) *P* < 0.01 (3) *P* < 0.01 (4) *P* < 0.05 (5) *P* < 0.05 (6) *P* < 0.01 (7) *P* < 0.01 (8) *P* < 0.01 (9) *P* < 0.01 (10) *P* < 0.01 (11) *P* < 0.01 (12) *P* < 0.01 (13) *P* < 0.01 (14) *P* < 0.01 (15) *P* < 0.01

A: average; BDNF: brain-derived neurotrophic factor; CST: corticospinal tract; d: day; EPO: erythroprotein; EPOR: erythroprotein receptor; g: gram; GAP-43: growth-associated protein 43; h: hour; icv: central venous injection; ig: intragastrical injection; IL: interleukin; IOD: integral optical density; ip: intraperitoneal injection; IV: infarct volume; iv: intravenous injection; kg: kilogram; LB: left behind; LF: left front; LFB: Luxol fast blue; MBP: myelin basic protein; MCAO: middle carotid artery occlusion; mg: milligram; NF-kBp65: nuclear transcription factors in rats Bp65; NFS: neurological function score; NGF: nerve growth factor; NR: not report; PCNA: proliferating cell nuclear antigen; pJAK2: phosphorylated janus kinase 2; pSTAT3: phosphorylated signal transducer and activator of transcription-3; RB: right behind; RF: right front; SD: Sprague Dawley; TTC: 2,3,5-triphenyltetrazolium chloride: VEGF: vascular endothelial growth factor; VWF: Von Willebrand factor; ZL: Zea Longa.

**Table 3:** Characteristics of mechanism studies of catalpol on experimental ischemic stroke.

Study (years)	Model	Method of administration (experimental group versus control group)	Observations	Possible mechanisms
Jia et al. [14]	MCAO/1 h in Wistar rats	Catalpol versus saline	Increased *β*-endorphin levels and the effects were reversed by GLP-1R orthosteric antagonist	GLP-1R/*β*-endorphin pathway
			Reduced IV and improved NFS	
Wan et al. [15]	Permanent MCAO in SD rats	Catalpol versus normal saline	Increased synapse quantity upregulated GAP43 expression	Promotion of survival, reparation, and regeneration of neural cells
			Improved NFS	
Zhu et al. [17]	Permanent MCAO in Kunming mice	Catalpol versus normal saline	Increased EPO and VEGF expression	Enhancement of angiogenesis
			Improved NFS, increased VWF and PCNA co-localization points, improved the vascular pattern of the cerebral cortex surface, and reduced BCEC edema	
Wang [18]	Permanent MCAO in SD rats	Catalpol versus normal saline	Upregulated the levels of VEGF, JAK2, and STAT3 Reduced IV	Enhancement of angiogenesis
Zhang et al. [20]	Permanent MCAO in Kunming mice	Catalpol versus no treatment	Increased SOD activity and decreased MDA and NO concentration Improved NFS	Reduction of oxidative reactions
Min et al. [21]	MCAO/2 h in SD rats	Catalpol versus dimethyl sulfoxide	Increased GSH-PX activity, decreasing MDA concentration Reduced IV and improved NFS	Reduction of oxidative reactions
Min [22]	MCAO/2 h in SD rats	Catalpol versus no treatment	Increased GSH-PX, SOD, and catalase activity, increased NOX2 expression, decreased MDA concentration Increased bcl-2 expression and decreased cleaved caspase-3 and Bax expression Reduced IV and improved NFS Reduced apoptotic cells	Reduction of oxidative reactions Inhibition of apoptosis
Liu 2011 [23]	MCAO/2 h in SD rats	Catalpol versus edible oil	Increased SOD activity, decreased MDA concentration Decreased IL10 expression Increased bcl-2 expression and decreased Bax expression Increased the expression of NGF, BDNF, VEGF, bFGF, TrKA, PI3K, and AKt, increased the mRNA levels of NGF, BDNF, TrKA, and AKt, decreased CDNF expression and PI3K mRNA level Decreased lactic acid content, increased pyruvic acid content, and improved Na^+^, K^+^-ATPase and Ca^2+^, Mg^2+^-ATPase activities Improved NFS Increased neuron number and nissl body number, improved cerebral cortex ultrastructure	Reduction of oxidative reactions Repression of inflammatory reactions Inhibition of apoptosis Promotion of survival, reparation, and regeneration of neural cells Relief of energy exhaustion
Wan et al. 2012 [24]	Permanent MCAO in SD rats	Catalpol versus normal saline	Increased dendritic branches and spine density and increased P38 expression Improved NFS	Promotion of survival, reparation, and regeneration of neural cells
Wang [25]	MCAO/2 h in SD rats	Catalpol versus normal saline	Increased LC3 expression Improved neurovascular unit structure Reduced IV and improved NFS	Repression of autophagy
Liu et al. 2011 [27]	Permanent MCAO in SD rats	Catalpol versus distilled water	Decreased lactic acid content, increased pyruvic acid content, and improved Na^+^, K^+^-ATPase and Ca^2+^, Mg^2+^-ATPase activities Improved NFS	Relief of energy exhaustion
Liu et al. 2011 [28]	Permanent MCAO in SD rats	Catalpol versus edible oil	Increased neuron number Increased the expression and mRNA levels of NGF and BDNF Improved NFS	Promotion of survival, reparation, and regeneration of neural cells
Wan et al. 2012 [31]	Permanent MCAO in SD rats	Catalpol versus normal saline	Increased dendritic branches and spine density Increased the expression of EPO, VEGF, STAT3, GAP-43, P38, BDNF, and Trk B Improved NFS	Enhancement of angiogenesis Promotion of survival, reparation, and regeneration of neural cells
Zhou [32]	Permanent MCAO in SD rats	Catalpol versus normal saline	Increased Ang-1 expression Improved NFS	Enhancement of angiogenesis
Wan et al. 2013 [33]	Permanent MCAO in SD rats	Catalpol versus normal saline	Increased remodeling and sprouting of CST axonal Increased the expression of GAP-43 Reduced IV and improved NFS	Enhancement of angiogenesis Promotion of survival, reparation, and regeneration of neural cells
Tan et al. [34]	Permanent MCAO in SD rats	Catalpol versus normal saline	Improved cell morphology and increased the number of neuron, glial cells, and vessel length Improved NFS	Enhancement of angiogenesis Promotion of survival, reparation, and regeneration of neural cells
Zhang et al. [35]	Permanent MCAO in SD rats	Catalpol versus edible oil and normal saline	Improved cell morphology and increased myelin sheath and increased MBP expression Improved NFS	Promotion of survival, reparation, and regeneration of neural cells
Qin [36]	Permanent MCAO in SD rats	Catalpol versus normal saline	Promote the proliferation and differentiation of neural stem cells and the survey of neuron Increased ratio of Bcl-2/Bax, reduced the expression of caspase-9 and caspase-3 Improved NFS	Inhibition of apoptosis
Wan et al. [38]	Permanent MCAO in SD rats	Catalpol versus no treatment	Increased VWF-PCNA colocalization Increased the expression of EPO, EPOR, pJAK2, pSTAT3, VEGF, and VEGF mRNA Improved NFS	Enhancement of angiogenesis

AKt: serine/threonine kinase; Ang-1: angiopoietin 1; BCEC: brain capillary endothelial cells; bcl-2: B-cell lymphoma-2; BDNF: brain-derived neurotrophic factor; bFGF: basic fibroblast growth factor; CDNF: cerebral dopamine neurotrophic factor; CST: corticospinal tract; EPO: erythroprotein; EPOR: erythroprotein receptor; GAP-43: growth-associated protein 43; GLP-1R: glucagon-like peptide-1 receptor; GSH-PX: glutathione peroxidase; HO-1: heme oxygenase-1; IL: interleukin; IV: infarct volume; JAK2: janus kinase 2; MCAO: middle carotid artery occlusion; MDA: malondialdehyde; NFS: neurological function score; NGF: nerve growth factor; PCNA: proliferating cell nuclear antigen; PI3K: phosphoinositide-3 kinase; pJAK2: phosphorylated janus kinase 2; pSTAT3: phosphorylated signal transducer and activator of transcription-3; SD: Sprague Dawley; SOD: superoxide dismutase; STAT3: the Stroke Therapy Academic Industry Roundtable; TrKA: tyrosine kinase receptor A; TrKB: tyrosine kinase receptor B; VEGF: vascular endothelial growth factor; VWF: Von Willebrand factor.

**Table 4:** Characteristics of mechanism studies of other compounds from Radix Rehmanniae on oxidation stress, anti-inflammation reactions, or apoptosis.

Study (years)	Model	Method of administration (experimental group versus control group)	Observations	Possible mechanisms
Liu [42]	Lipid peroxidation in rat liver microsome induced by Fe^2+^-cysteine	Rehmaglutoside E versus no treatment	Decreased MDA content	Reduction of oxidative reactions
	Lipid peroxidation in rat liver microsome induced by Fe^2+^-cysteine	6-O-E-Caffeoyl ajugol versus no treatment	Decreased MDA content	Reduction of oxidative reactions
	(1) Lipid peroxidation in rat liver microsome induced by Fe^2+^-cysteine (2) Inflammation in cells induced by LPS	Leucosceptoside A versus no treatment	(1) Decreased MDA content (2) Decreased NO production	Reduction of oxidative reactions, repression of inflammatory reactions
	Lipid peroxidation in rat liver microsome induced by Fe^2+^-cysteine	Jionoside D versus no treatment	Decreased MDA content	Reduction of oxidative reactions
	Inflammation in cells induced by LPS	Acteoside versus no treatment	Decreased NO production	Repression of inflammatory reactions
	Inflammation in cells induced by LPS	Salidrosid versus no treatment	Decreased NO production	Repression of inflammatory reactions
	Inflammation in cells induced by LPS	Jionoside D versus no treatment	Decreased NO production	Repression of inflammatory reactions
	Inflammation in cells induced by LPS	Jionoside B1 versus no treatment	Decreased NO production	Repression of inflammatory reactions
	Inflammation in cells induced by LPS	Vanillin versus no treatment	Decreased NO production	Repression of inflammatory reactions
Nan et al. [43]	Inflammation in mouse microglial cells induced by LPS	Ajugol versus no treatment	Decreased NO production	Repression of inflammatory reactions
Chai et al. [44]	Bile duct-ligated SD rats	Oleanolic acid versus saline	Decreased serum TNF-*α*, IL-1*β*, and IL-6	Repression of inflammatory reactions
			Decreased serum TBA, TBIL, DBIL, ALP, ALT, and AST Reduced serum total bile acid and bile salt	
Goyal et al. [45]	Cardiac toxicity rats induced by doxorubicin	Oleanolic acid versus no treatment	Decrease the activities of GSH, SOD, and catalase and MDA level	Reduction of oxidative reactions
			Decreased CK-MB, LDH, and heart weight Improved alterations in ECG and histopathology of myocardium	
Liu et al. [46]	Oxidative damage in PC12 cells induced by hydrogen peroxide	Geniposide versus no treatment	Increased the expression of Bcl-2 and HO-1, delayed the peak of cAMP level	Reduction of oxidative reactions
			Decreased apoptotic and necrotic cells and increased the viability of PC12 cells	Inhibition of apoptosis
Wang et al. [47]	(1) MCAO/2 h in SD rats (2) Oxygen-glucose deprivation/4 h in primary microglial cell	Geniposide versus no treatment	(1) Reduced infarct volume and inhibited the activation of microglial cells in ischemic penumbra (2) Decreased cell viability, the secretion of TNF-*α*, IL-1*β*, IL-6, IL-8, and IL-10, the expression of TLR4, and NF-kBp65, TLR4 mRNA level, nuclear translocation of NF-kBp65	Repression of inflammatory reactions

ALP: alkaline phosphatase; ALT: alanine aminotransferase; AST: aspartate aminotransferase; bcl-2: B-cell lymphoma-2; cAMP: cyclic adenosine monophosphate; CK-MB: creatine kinase isoenzyme-MB; DBIL: direct bilirubin; ECG: electrocardiograph; GSH: glutathione; HO-1: heme oxygenase-1; IL: interleukin; LDH: lactate dehydrogenase; LPS: lipopolysaccharide; MCAO: middle carotid artery occlusion; MDA: malondialdehyde; NF-kBp65: nuclear transcription factors in rats Bp65; SD: Sprague Dawley; SOD: superoxide dismutase; TBA: total bile salts; TBIL: total bilirubin; TLR4: toll-like receptor 4; TNF-*α*: tumor necrosis factor-*α*.

## Abbreviations

AKt: Serine/threonine kinase
Ang-1: Angiopoietin 1
ARRIVE: The animal research: reporting in vivo experiments
bcl-2: B-cell lymphoma-2
BDNF: Brain-derived neurotrophic factor
bFGF: Basic fibroblast growth factor
CAMARADES: Collaborative Approach to Meta-Analysis and Review of Animal Data from Experimental Studies
CDNF: Cerebral dopamine neurotrophic factor
CNKI: National Knowledge Infrastructure
df: Degrees of freedom
EMBASE: Excerpta Medica Database
EPO: Erythroprotein
EPOR: Erythroprotein receptor
GAP-43: Growth-associated protein 43
GLP-1R: Glucagon-like peptide-1 receptor
GSH-PX: Glutathione peroxidase
HO-1: Heme oxygenase-1
IL: Interleukin
IV: Infarct volume
JAK2: Janus kinase 2
KDR: Kinase insert domain-containing receptor
ALP: Alkaline phosphatase
MCAO: Middle carotid artery occlusion
MDA: Malondialdehyde
NFS: Neurological function score
NGF: Nerve growth factor
NOX2: Nicotinamide adenine dinucleotide 2′-phosphate oxidase 2
PCNA: Proliferating cell nuclear antigen
Ph.D.: Philosophiae Doctor
PI3K: Phosphoinositide-3 kinase
pJAK2: Phosphorylated janus kinase 2
SD: Sprague Dawley
SMD: Standard mean difference
SOD: Superoxide dismutase
STAIR: The Stroke Therapy Academic Industry Roundtable
STAT3: Signal Transducer and Activator of Transcription 3
TrKA: Tyrosine kinase receptor A
TrKB: Tyrosine kinase receptor B
TTC: 2,3,5-Triphenyltetrazolium chloride
VEGF: Vascular endothelial growth factor
VWF: Von Willebrand factor
ZL: Zea Longa.
